# A novel crosstalk between CCAR2 and AKT pathway in the regulation of cancer cell proliferation

**DOI:** 10.1038/cddis.2016.359

**Published:** 2016-11-03

**Authors:** Michela Restelli, Martina Magni, Vincenzo Ruscica, Patrizia Pinciroli, Loris De Cecco, Giacomo Buscemi, Domenico Delia, Laura Zannini

**Affiliations:** 1 Department of Experimental Oncology, Fondazione IRCCS Istituto Nazionale dei Tumori, Via Amadeo 42, Milan 20133, Italy; 2 Department of Biochemistry, Max Planck Institute for Developmental Biology, Tubingen, Germany; 3 Department of Biosciences, University of Milan, via Celoria 26, Milan 20133, Italy

## Abstract

Human CCAR2 has recently emerged as having a pivotal role in the DNA damage response, promoting apoptosis and repair of heterochromatic DNA breaks. However, less is known about the function of CCAR2 in tumor formation and cancer progression. Here, we demonstrate, for the first time, that CCAR2 loss inhibits the proliferation of cancer cells, but preserves the growth of normal cells. Investigating the mechanisms responsible for this differential effect, we found that CCAR2 depletion specifically impairs the activation of AKT pathway in cancer cells, but not in normal cells, by reducing AKT phosphorylation on Ser473. This effect is achieved through the transcriptional upregulation of *TRB3* gene and accumulation of TRB3 protein, which then binds to and inhibits the phosphorylation and activation of AKT. The defective activation of AKT finally results in reduced GSK3*β* phosphorylation, prevention of G1/S transition and inhibition of cancer cell growth. These results establish an important role for CCAR2 in cancer cells proliferation and could shed new light on novel therapeutic strategies against cancer, devoid of detrimental side effects.

Human CCAR2 (cell cycle and apoptosis regulator 2, also known as DBC1) is a nuclear protein involved in various biological processes including DNA damage response, metabolism, epigenetics, nuclear receptor function, circadian cycle, mRNA splicing,^1^ and B cell development.^2^ We previously demonstrated that CCAR2, phosphorylated by ATM/ATR and cooperating with the checkpoint kinase Chk2 and the proteasome subunit REG*γ*, negatively regulates the NAD^+^-dependent histone deacetylase SIRT1, promoting p53 activation and apoptosis induction in presence of DNA damage.^3, 4^ Recently, we also reported that CCAR2 itself modulates the activity of Chk2 towards KRAB-associated protein 1 (KAP1), thus influencing the repair of DNA breaks.^5^

However, although the role of CCAR2 in the DNA damage response is well established, its function in tumor formation and cancer progression is still controversial. Originally named DBC1 by mistake because its gene maps to a region of chromosome 8 that was found homozygously deleted in breast cancers,^1^ CCAR2 was instead upregulated in many types of tumors.^1^ These findings, together with the fact that CCAR2 loss *in vitro* prevents cancer cells growth^6, 7^ suggested for this protein a role of tumor promoter. On the other hand, CCAR2-knockout mice are tumor prone^8^ and, intriguingly, in some cancer patients, CCAR2 down-regulation is associated with poor prognosis,^1^ suggesting that it acts as tumor suppressor.

The PI3K-AKT pathway has a major role in regulating cellular processes that are hallmarks of cancer cells, like proliferation, survival or migration. This signaling cascade is frequently hyperactivated in human malignancies^9, 10^ thus representing an attractive target for cancer therapy. Indeed inhibitors of the PI3K-AKT pathway are being tested in clinical trials.^11^ PI3Ks constitute a family of lipid kinases that, in response to growth factor stimulation, are phosphorylated and activated by cell surface receptor tyrosine kinases, such as platelet-derived growth factor receptor, insulin-like growth factor 1 receptor and epidermal growth factor receptor. Once activated, PI3Ks catalytic subunit converts the substrate phosphatidylinositol 4,5-biphosphate into phosphatidylinositol-3,4,5-triphosphate on the plasma membrane, which in turn recruits signaling proteins including the phosphoinositide-dependent kinase-1 and AKT.^10^ AKT is activated by phosphorylation at threonine 308 (Thr308) and at serine 473 (Ser473). Once activated, AKT phosphorylates a large number of downstream targets, which regulate cell growth and protein synthesis, increasing proliferation and cell cycle progression.^12^ AKT activity is negatively regulated by the phosphatase and tensin homolog PTEN that, by dephosphorylating phosphatidylinositol-3,4,5-triphosphate, blocks AKT recruitment to the cell membrane finally resulting in the inhibition of AKT signaling pathway.^13^ Among the inhibitors of AKT activation there is also the protein TRB3, a pseudokinase characterized by a kinase-like domain lacking the conserved catalytic residues.^14^ Specifically, TRB3 inhibits AKT activation by binding to this kinase and blocking its phosphorylation on Ser473 by mTORC2.^15^ Accordingly it was also reported that administration of different anti-cancer agents promotes cancer cell death via TRB3 upregulation and the subsequent inhibition of AKT.^16^

Here, we show for the first time that CCAR2, TRB3 and AKT are linked together in a regulatory pathway that controls cancer cell proliferation and leaves unaffected the growth of non malignant cells. In particular, we demonstrate that CCAR2 loss causes a strong reduction of cancer cell proliferation associated with a significant increase of TRB3 at both transcript and protein level. Finally, augmented TRB3 promotes the inhibition of AKT activation and G1/S transition.

## Results

### CCAR2 depletion impairs U2OS cell proliferation by altering the AKT pathway

**Figure 1:**
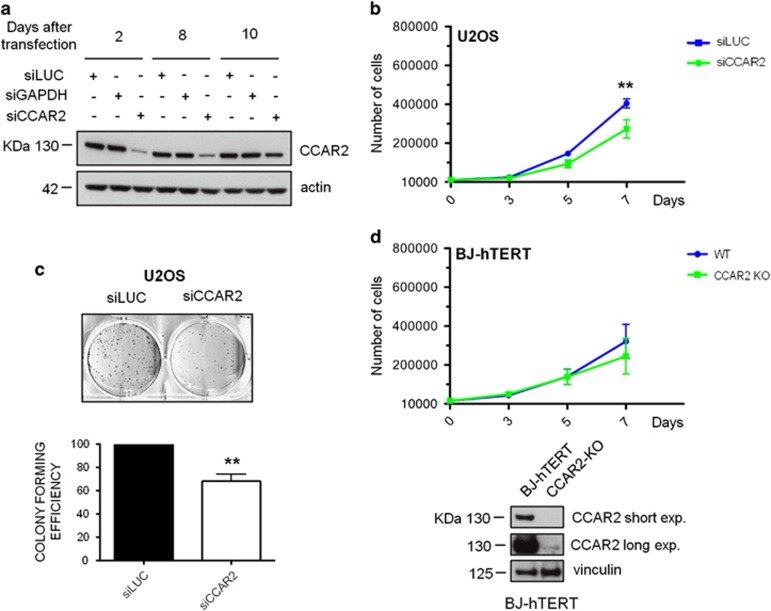
CCAR2 depletion strongly inhibits U2OS cell proliferation. (**a**) Western blot (WB) analysis demonstrating CCAR2 depletion for up to 10 days after siRNA transfection. (**b**) Cell proliferation rate in control and CCAR2 silenced U2OS cells. **P<0.01. (**c**) Colony forming assays in U2OS cells silenced for CCAR2 (top). Colonies were counted and expressed as a percentage of siLUC control. Results are mean +/− S.D. from three inedependent experiments. **P<0.01 (**d**) Cell proliferation rate (top) and WB analysis (bottom) of BJ-hTERT control cells and CCAR2-KO mass culture

To evaluate the effect of CCAR2 depletion on cellular proliferation, U2OS cells were transfected with a pool of four different CCAR2 iBONi siRNAs (RIBOXX), which provide an effective silencing for up to 10 days (Figure 1a), and the growth ability and colony forming efficiency was evaluated at different time points. Depletion of CCAR2 induced a significant decrease in U2OS cell proliferation and colony formation (Figures 1b and c). Conversely, a similar analysis performed with a population of BJ-hTERT-KO cells^5^ revealed no proliferation defects (Figure 1d). Collectively these results demonstrate that CCAR2 loss impairs the growth of U2OS, but not of BJ-hTERT cells.

**Figure 2:**
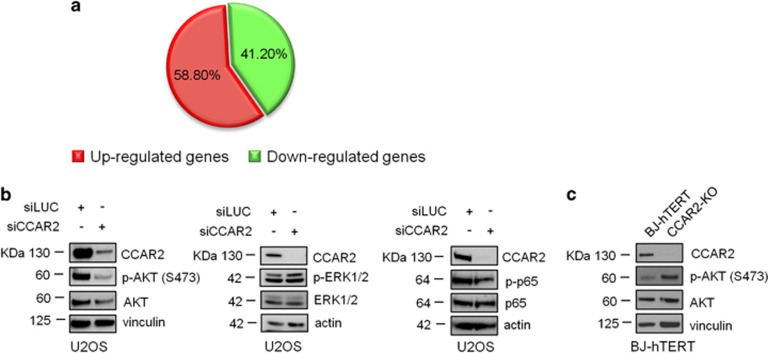
CCAR2 depletion reduces AKT phosphorylation. (**a**) Pie chart indicating the percentage of up- and downregulated genes identified by the gene expression analysis. (**b**) WB analysis of U2OS cells silenced for CCAR2 with the indicated antibodies. (**c**) WB analysis of control and CCAR2-KO mass culture BJ-hTERT cells with the indicated antibodies

To investigate the mechanism underlying the regulation of U2OS cells growth by CCAR2, we performed a genome-wide gene expression analysis in control (siLUC) and CCAR2 silenced U2OS cells 6 days after transfection (Supplementary Figure 1). Microarray analysis identified 165 CCAR2 regulated genes that exhibited a fold change >1.5 between siLUC and siCCAR2 transfected cells. Overall, 97 of these genes (58.8%) were found to be upregulated, whereas 68 (41.2%) were downregulated (Figure 2a; Supplementary Table 1). To gain insights into the function of CCAR2 regulated genes, we carried out an ingenuity pathway analysis (IPA) and found that among the five top molecular and cellular functions, ‘Cellular growth and proliferation' and ‘Cell death and survival' were included (Supplementary Table 2). In addition, we identified three networks strongly altered in CCAR2 silenced cells, compared with controls, named N1, N2 and N3 (Supplementary Figures 2A–C). All these pathways are involved in sustaining cancer cell growth and N1 has NF-kB as the hub gene, N2 includes several genes regulating AKT kinase and N3 is linked to ERK1/2 pathway. A similar analysis was also performed between parental BJ-hTERT and the mass culture of BJ-hTERT-KO for *CCAR2* gene, that does not have growth defects, but none of these pathways was found to be affected (Supplementary Table 3). To further corroborate these results, we silenced CCAR2 in U2OS cells and evaluated its effect on NF-kB, AKT and ERK1/2 activation. Compared with the control, CCAR2 silencing (Figure 2b) determined a reduction in AKT phosphorylation on S473 (a hallmark of its activation), whereas it left unaltered ERK1/2 and NF-kB. On the other hand, CCAR2 depletion in BJ-hTERT cells induced AKT activation (Figure 2c).

Altogether these results suggest that CCAR2 loss negatively impacts on AKT activation only in cancer cells.

### CCAR2 specifically regulates the proliferation of human cancer cell lines

**Figure 3:**
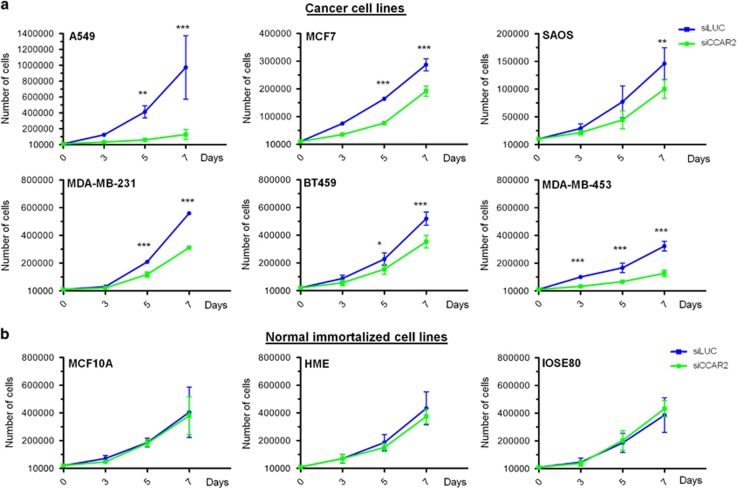
CCAR2 depletion decreases cancer cell proliferation but preserves the growth of normal cells. Proliferation rate analyses in the indicated cancer (**a**) and normal immortalized (**b**) cell lines silenced for CCAR2 and siLUC as negative control. *P<0.05, **P<0.01, ***P<0.001

**Figure 4:**
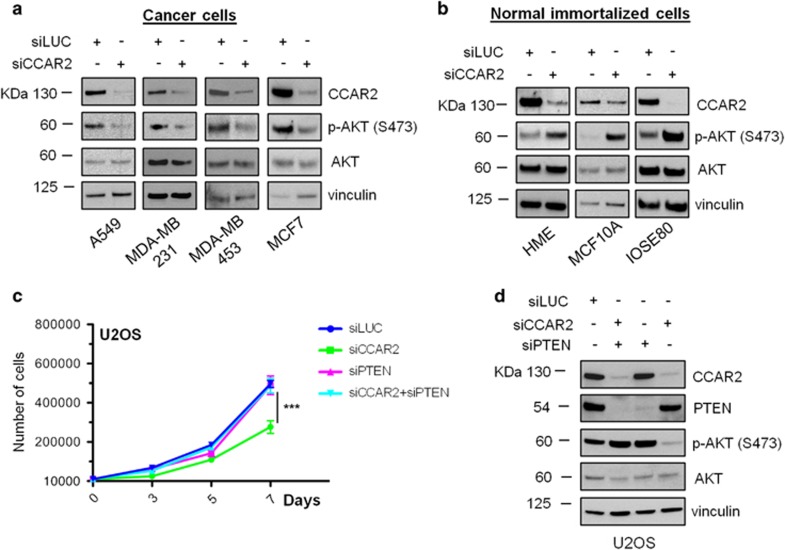
CCAR2 depletion reduces AKT activation in cancer cells, but increases phosphorylated AKT in normal cells. WB analyses of AKT phosphorylation in the indicated cancer (**a**) and normal immortalized (**b**) cell lines silenced for CCAR2. (**c**) Cell proliferation rate in U2OS cells silenced for CCAR2, PTEN or both CCAR2 and PTEN. ***P<0.001 (**d**) WB analysis with the indicated antibodies of U2OS cells transfected with siRNA against CCAR2, PTEN and both siCCAR2 and siPTEN

CCAR2 loss was reported to specifically reduce the growth of cancer cells with mutant p53^8^ and negatively affect the proliferation of estrogen receptor (ER) negative cells,^17^ whereas its role in ER positive cells growth is disputed.^18, 19^ Therefore, we extended the analysis of CCAR2 silencing effects to a panel of several cancer cell lines with different p53 and ER status (Supplementary Table 4A and B), and we intriguingly found that CCAR2 depletion strongly inhibited the proliferation of lung, osteosarcoma and breast cancer cells (A549, SAOS2, MCF-7, MDA-MB-231, BT549, and MDA-MB-453) regardless of p53 and ER status (Figure 3a; Supplementary Figures 3A and C). Conversely, as for BJ-hTERT, CCAR2 silencing had no effect on the growth of non malignant cells (like MCF10A, HME and IOSE80) (Figure 3b; Supplementary Figures 3B and C). We next verified the effect of CCAR2 knock-down on AKT activation and interestingly found a correlation in all cancer cells between growth inhibition and decreased AKT phosphorylation (Figure 4a). On the other hand, the lack of growth defects in the CCAR2-depleted normal cells was associated with increased levels of phosphorylated AKT (Figure 4b).

To evaluate the specificity of this phenomenon, we transfected A549 and HME cells with other two siRNA sequences or with their combination and we evaluated their effect on cellular proliferation and AKT phosphorylation. We found that all the tested siRNAs induce a proliferation defect associated with reduced AKT phosphorylation in A549, but not in HME cells (Supplementary Figures 4A and B).

To prove that the proliferation defect caused by CCAR2 depletion depends on reduced AKT activation, U2OS and A549 cells were silenced for CCAR2 and PTEN, to mimic constitutive AKT activation, and their effect on cellular proliferation and AKT phosphorylation was verified. We demonstrate that PTEN depletion rescued the growth defect induced by CCAR2 depletion in U2OS and, partially, in A549 cells and restored the phosphorylation of AKT in both cell lines (Figures 4c and d; Supplementary Figures 5A and B). Moreover, we silenced AKT and CCAR2 in A549 and HME cells, and we found that loss of both proteins does not worsen the anti-proliferative effect of CCAR2 depletion alone in A549, but reduces the growth of HME cells (Supplementary Figures 6A and B). These data indicate that CCAR2 and AKT have epistatic roles in cancer cells.

Furthermore, we evaluated if SIRT1, the major CCAR2 target,^20^ is implicated in this CCAR2 function. As shown in Supplementary Figure 7, the depletion of SIRT1 did not rescue the growth defect induced by CCAR2 silencing in A549 cells.

All these data indicate that CCAR2 depletion inhibits selectively the proliferation of cancer cells, in a SIRT1 independent manner, by reducing AKT activation.

**Figure 5:**
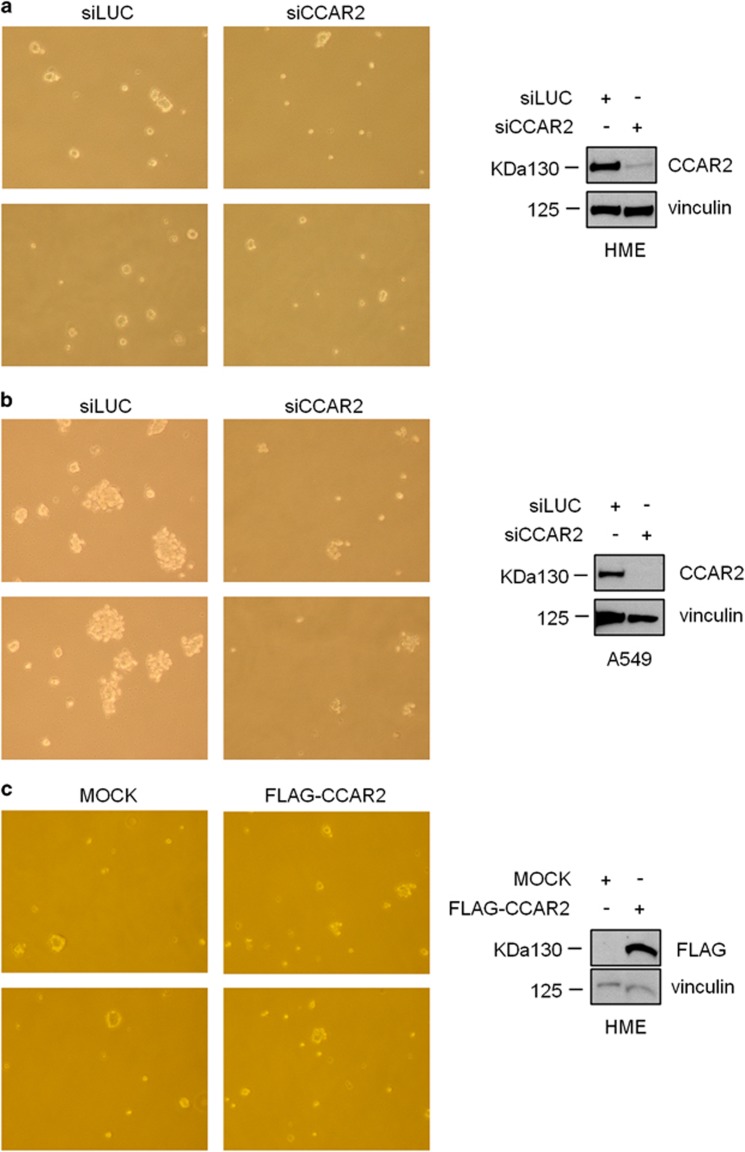
CCAR2 protein levels do not affect malignant transformation. Representative images of colonies formed in soft agar by HME (**a**) and A549 (**b**) cells silenced for CCAR2. (**c**) Representative images of HME colonies formed in soft agar by a clone stably expressing FLAG-CCAR2

To better evaluate the biological effect of CCAR2 silencing, we performed soft agar growth assays with HME and A549 cells. We found that CCAR2 depletion, does not increase the ability to grow in soft agar of HME cells, despite the induction of AKT phosphorylation, whereas, as expected, reduces the anchorage independent growth of A549 cells (Figures 5a and b). We also tested if CCAR2 overexpression can induce malignant transformation and found, with a stably transfected clone, that ectopic CCAR2 does not increase the growth in soft agar of HME cells (Figure 5c). Altogether these results indicate that alteration of CCAR2 protein levels cannot induce malignant transformation of normal cells.

Finally, we tested if CCAR2 loss can increase the sensitivity of cancer cells to anti-cancer agents. For this A549 and HME cells were silenced for CCAR2 and their viability was analyzed 48 h after etoposide or tamoxifen treatment. As evidenced in the charts, CCAR2 depletion protected cancer cells from etoposide induced cell death, but increased their sensitivity to tamoxifen (Supplementary Figure 8).

### CCAR2 depletion affects S-phase progression

**Figure 6:**
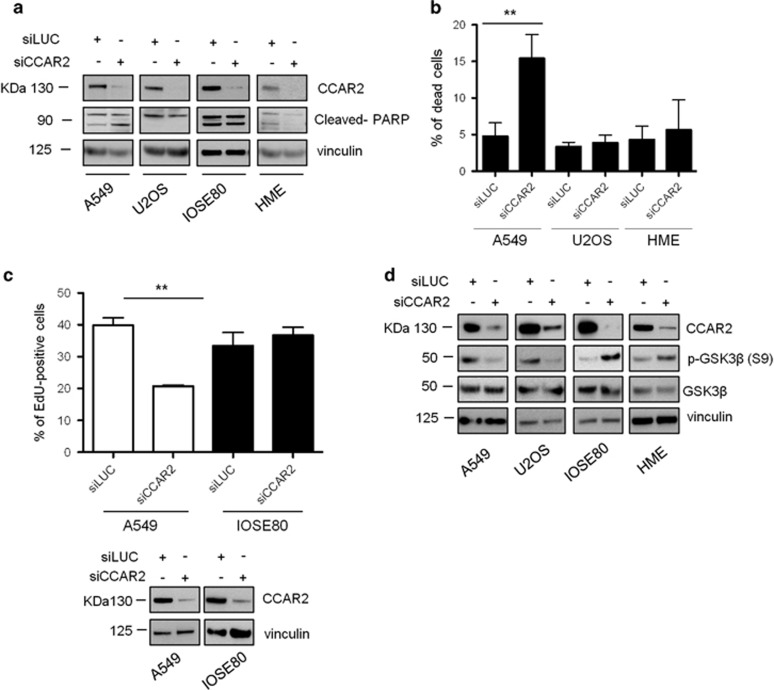
CCAR2 depletion prevents G1/S-phase transition in cancer cell lines. Normal and cancer cells silenced for CCAR2 were analyzed by western blot for the levels of cleaved PARP protein (**a**) and by trypan blue exclusion test for the percentage of dead cells (**b**). (**c**) A549 and IOSE80 cells transfected with siCCAR2 and siLUC were incubated in EdU containing medium for 1 h 30 min. EdU-positive cells were stained, enumerated and data reported in the chart (top). **P<0.01 WB analysis of A459 and IOSE80 cells transfected with siCCAR2 and siLUC, with the indicated antibodies (bottom). (**d**) WB analysis of cancer (U2OS and A549) and normal immortalized cells (HME and IOSE80) silenced for CCAR2 with the indicated antibodies

To identify the mechanism responsible for the reduced proliferation of CCAR2-depleted cancer cells, we initially analyzed the levels of the apoptotic marker cleaved PARP, but no significant differences between control and CCAR2-depleted cells were found except for A549, where a slight increase of cleaved PARP levels was detectable in CCAR2 silenced cells (Figure 6a). Moreover, we evaluated the percentage of dead cells by trypan blue exclusion test in HME, U2OS and A549 cells depleted of CCAR2 and also in this case we confirmed that CCAR2 absence does not affect the viability of HME and U2OS cells, whereas it rises the percentage of dead cells in A549 from about 5–15% (Figure 6b). As these differences cannot account for the growth defect detected in cancer cells, we analyzed the effect of CCAR2 silencing on cell cycle progression. We labeled A549 cells with the 5-bromo-2′-deoxyuridine analog EdU, which marks S-phase cells^8^ and found that the fraction of EdU-positive cells accounted for 40% in siLUC controls and only about 20% in CCAR2 silenced cells (Figure 6c). Notably, no differences in EdU positivity were found between siLUC and siCCAR2 in the normal cell line IOSE80 (Figure 6c).

To further ascribe the defect in G1/S transition to the reduced AKT activation, we analyzed in different cell lines the phosphorylation of GSK3*β*, whose phosphorylation by AKT is required to allow G1/S progression.^12^ In accordance with our hypothesis, we found that the phosphorylation of GSK3*β* on Ser9 is reduced in A549, U2OS and MDA-MB-231 cells depleted of CCAR2, but induced in silenced HME and IOSE80 normal cells (Figure 6d). Altogether these results suggest that CCAR2 depletion reduces AKT activation and consequently GSK3*β* phosphorylation, finally preventing G1/S transition in cancer cells.

As these data contrast with those we previously published about normal cell cycle progression of CCAR2-KO U2OS clones,^5^ we analyzed proliferation and AKT phosphorylation in these cells, but we did not find defects in AKT activation or in the rate of proliferation (Supplementary Figure 9), probably reflecting the acquired ability of CCAR2-KO clones to counteract the absence of CCAR2.

### CCAR2 inhibits AKT activation by regulating TRB3 expression

To understand the molecular mechanism underlying CCAR2-dependent regulation of AKT activation, we performed co-immunoprecipitation analyses in both A549 and MCF10A cells, but we were unable to find any CCAR2-AKT association (Supplementary Figure 10).

**Figure 7:**
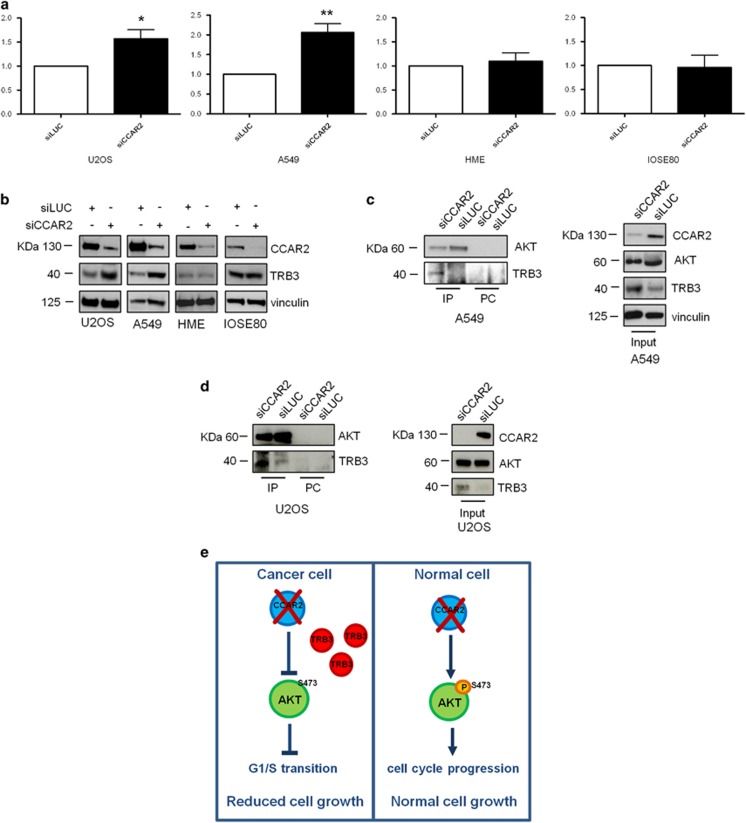
CCAR2 depletion induces the expression of *TRB3* in cancer cell lines. (**a**) Expression of *TRB3* was analyzed by RT-qPCR in cancer (U2OS and A549) and normal cells (HME and IOSE80) transfected with siCCAR2 and siLUC. *P<0.05, **P<0.01. (**b**) WB analyses of cancer and normal immortalized cells transfected with siCCAR2 and siLUC with the indicated antibodies. AKT immunoprecipitates from A549 (**c**) and U2OS (**d**) cells silenced for CCAR2 and LUC were analyzed by WB with the indicated antibodies. PC= negative control. (**e**) Graphical representation of our working model

Thus, since our gene expression profile analysis revealed that in U2OS CCAR2 altered the expression of genes related to the AKT pathway, we performed RT-qPCR analysis in different cell lines silenced for CCAR2, with a focus on *TRB3,* since alteration in its expression is known to impact on AKT phosphorylation and activation.^14, 15^ We found that CCAR2 depletion induces the accumulation of TRB3 mRNA and protein in U2OS and A549 cells, but not in the normal cell lines HME and IOSE80 (Figures 7a and b). As TRB3 is known to bind and inhibit AKT,^14, 15^ we performed co-immunoprecipitation analyses in control and CCAR2 silenced cells, and we found that the association between AKT and TRB3 increases upon CCAR2 depletion in A549 and U2OS cells (Figures 7c and d), but is not modulated in HME and IOSE80 cells (Supplementary Figures 11A and B).

Collectively these results indicate that, in cancer cells, CCAR2 loss induces the accumulation of TRB3 leading to an augmented binding and inhibition of AKT.

## Discussion

Our studies demonstrate a critical role for CCAR2 in cancer cell proliferation, where its depletion negatively impacts on their growth ability, but they also interestingly show, for the first time, that CCAR2 absence preserves the proliferation of normal cells. This phenomenon suggests that cancer cells, owing to accumulated alterations during the transformation process, may become addicted to CCAR2 expression, and its ablation may establish a synthetic lethality effect.

To understand this phenomenon, we performed gene expression profiles and found, specifically in CCAR2-deficient cancer cells, a severe perturbation of the AKT pathway, which is frequently hyperactivated in several human tumors and in cancer cell lines.^21^ Accordingly, we discovered that CCAR2 depletion determines growth reduction and impairment of AKT activation in all the tested cancer cells, even if to a different extent because of the diverse growth rate of the analyzed cell lines. This growth defect finally results in a reduced ability to grow in soft agar and increased sensitivity to tamoxifen treatment, but not to etoposide, which is known to induce CCAR2-SIRT1 association and apoptosis inhibition.^3^ By contrast, in non malignant cells, CCAR2 silencing had no effect on proliferation and was associated with increased AKT phosphorylation that, however, does not contribute to malignant transformation as both CCAR2 depletion and overexpression does not allow the anchorage independent growth of normal cells. Thus, it is possible that cells require AKT activation to survive CCAR2 loss and that only cells with an intact AKT pathway can do it. However, further experiments are necessary to determine the reason why CCAR2 has this effect specifically in cancer cells and not in normal cells. One hypothesis is that CCAR2 depletion could lead to a synthetic lethal effect with the hyperactivation of AKT, mainly detectable in cancer cells. Alternatively, CCAR2 could interact with some transcriptional regulators and modulate their activity differently in cancer and normal cells. Accordingly, the expression of genes implicated in the induction of AKT function, such as MSR1 and RAB11-FIP1,^22, 23^ has been found deregulated in BJ-hTERT cells and, on the contrary, genes involved in AKT repression are specifically altered in U2OS cells. Another possibility is that CCAR2 loss, which is known to induce metabolic defects,^24^ could be more detrimental in cancer than in normal cells because of the Warburg effect and because of normal cells ability to activate AKT also in the absence of CCAR2. In accordance with this hypothesis, we found that TRB3, whose gene transcription is induced by aminoacids deprivation,^25^ and which is known to inhibit AKT activation,^15, 16^ is upregulated in cancer cells depleted of CCAR2, but not in normal cells. Therefore, we demonstrated that the inhibition of AKT activity by CCAR2 depletion in cancer cells could be, at least in part, due to the upregulation of *TRB3* gene, which results in TRB3 protein accumulation and increased interaction with the AKT protein. Differently, the inability to induce TRB3 accumulation, could explain the reason why in normal cells CCAR2 depletion can promote AKT phosphorylation. Unfortunately, we were not able to perform proliferation assays in cells silenced for CCAR2 and TRB3, because of the reported toxic effect of TRB3 depletion.^26, 27^ Anyway, other genes encoding for proteins related to the AKT pathway could also be implicated as suggested by our results and previous reports. Indeed, we found that in CCAR2-depleted cells also the expression of *IGFBP5*, *PPARG* and PDGFA is altered and all these proteins can directly or indirectly regulate AKT activation.^16, 28, 29, 30^ Moreover, it was previously shown that CCAR2 regulates breast and colon cancer progression by acting respectively as PEA3 and PROX-1 transcription factors co-activator^7, 17^ and, of note also these proteins are implicated in AKT pathway regulation.^31, 32^

Finally, our studies suggest a model (Figure 7e) in which CCAR2 depletion in cancer cells promotes the inactivation of the AKT pathway by altering the transcription of genes coding for proteins implicated in the regulation of AKT activity, such as TRB3. AKT inactivation then leads to reduced phosphorylation of GSK3*β*, which in turn prevents G1/S-phase progression and cells proliferation. In normal cells, however, CCAR2 depletion leads to AKT activation and leaves unaltered the cellular growth. However, further studies will be necessary to elucidate the mechanisms responsible for this differential behavior of CCAR2 in normal and cancer cells and will provide useful insights for the discovery of novel therapeutic targets for the treatment of multiple cancers.

## Materials and Methods

### Cell lines

Human osteosarcoma U2OS and SAOS cells, human lung cancer A549 cells, breast cancer MCF-7, BT549 and MDA-MB-453 cells were grown in DMEM (Lonza, Basel, Switzerland) with 10% of fetal bovine serum (FBS). Breast cancer MDA-MB-231 and the normal ovarian IOSE80 cells were cultured in RPMI-1640 (Lonza) with 10% FBS. BJ-hTERT human fibroblast cells were grown in DMEM/Medium199 (4:1) with 10% of fetal bovine serum and 10 *μ*g/ml Hygromycin B. Human non tumorigenic mammary epithelial MCF10A and HME cells were cultured in DMEM/F12 (1:1, Lonza) with 10% of FBS, insulin (5 *μ*g/ml), hydrocortisone (1 *μ*g/ml), and human epidermal growth factor (hEGF; 10 ng/ml).

### Expression vectors, siRNAs and transfections

siRNAs against CCAR2 were iBONi siRNA Pool (RIBOXX, Radebeul, Germany) or single sequences. siRNA against SIRT1 were ON-TARGET plus SMART pool (GE Healthcare Dharmacon, Lafayette, CO, USA), whereas those against PTEN and AKT were FlexiTube siRNA (Qiagen, Valencia, CA, USA). Lipofectamine 2000 (Thermo Fisher Scientific, Waltham, MA, USA) and Lipofectamine RNAiMAX (Thermo Fisher Scientific) were used for plasmids and siRNAs transfections, respectively, according to the manufacturer's instructions.

### Cell growth analysis

The day after control or CCAR2 siRNA transfection, cells were seeded in a 12 wells plate at 10 000 cells per well (for MCF10A 20000 cells were seeded) in duplicate. Grown cells were then counted at the indicated days and results of three independent experiments were reported in the chart.

To test the sensitivity to anti-cancer agents, cells transfected with CCAR2 siRNA were seeded in a 96 wells plate and, 24 h later, treated with etoposide or tamoxifen at the indicated concentrations. Viability was determined 48 h after treatment by CellTiter-Glo Cell Viability Luminescent Assay (Promega, Madison, WI, USA) according to manufacturer's procedures.

### Soft agar assay

Soft agar assays were performed as previously described.^33^ Briefly, 5 × 10^4^ cells were suspended in 1.5 ml of the appropriate complete medium containing 0.33% agar, added into a layer of complete medium containing 0.5% agar and incubated for 3 weeks. Plates were then analyzed for colony number and size and representative images were captured by optical microscope.

### Western blot, antibodies and immunoprecipitations

The NuPAGE system (Thermo Fisher Scientific) was used for western blot analyses.^4^ Quantification of protein levels were normalized to loading control and for phosphorylated proteins to total proteins. Proteins were transferred to a PVDF membrane (Protran, Merck Millipore, Darmstadt, Germany). The blots were incubated with the following antibodies: CCAR2 (Bethyl Laboratories, Montgomery, TX, USA or Cell Signaling Technology, Danvers, MA, USA); phospho-AKT-S473 (Cell Signaling Technology), pan-AKT (Cell Signaling Technology), SIRT1 (Cell Signaling Technology), PTEN (Cell signaling Technology), Vinculin (Sigma-Aldrich, Darmstadt, Germany) and *β*-Actin (Sigma). For immunoprecipitation analyses, A549 and MCF10A cells were lysed with ELB buffer^3^ and 1 mg of total protein extracts were incubated with anti-CCAR2 antibody (Bethyl Laboratories) for 4 h at 4 °C. Beads were washed three times with ELB buffer and then resuspended in Laemmli buffer heated at 98 °C, loaded on NuPAGE gels and subjected to western blot analyses with the indicated antibodies.

### Microarray analyses

Total RNA was isolated from U2OS and BJ-hTERT samples using Qiazol (Qiagen, Valencia, CA, USA) reagent. After a clean-up treatment with RNAeasy kit following the manufacture's recommendations (Qiagen) and with RNase-free DNase to remove contaminating genomic DNA, RNA integrity and purity was assessed by Bioanalyzer (Agilent Technology, Santa Clara, CA, USA).

RNA samples were processed for microarray hybridization by the Functional Genomics core facility at the Fondazione INT (Milan). Briefly, 300 ng of total RNA was reverse transcribed, labeled with biotin and amplified overnight (14 h) using the Illumina RNA TotalPrep Amplification kit (Ambion, Thermo Fisher Scientific, Waltham, MA, USA) according to manufacturer's protocol. Overall, 1 *μ*g of the biotinylated cRNA sample were mixed with the Hyb E1 hybridizatioin buffer containing 37.5% (w/w) formamide and then hybridized to Sentrix Bead Chip Human HT12_v4 (Illumina, San Diego, CA, USA) at 58 °C overnight (18 h). The array represents over 47 000 bead types, each with a unique sequence derived from human genes in the National Centre for Biotechnology Information Reference Sequence and UniGene database. Array chips were washed with manufacturer's E1BC solution, stained with 1 *μ*g/ml Cy3-streptavidine (Amersham Biosciences) and eventually scanned with Illumina BeadArray Reader. We collected primary data using the supplied scanner software and GenomeStudio v2009.1 software package (Illumina, San Diego, CA, USA).

Microarray data were analyzed through the R programming language (v 3.0.0, http://www.R-project.org/). All samples passed quality controls; raw data were log_2_ transformed and normalized using the robust spline normalization (RSN) method implemented in the *lumi*^34^ package of Bioconductor.^35^ Probes with a detection *P*-value<0.01 in at least 1 sample were included in the statistical analysis; in case of multiple probes targeting the same gene symbol, the probe detected in the highest number of samples was selected (the probe with the highest interquartile range was chosen in case of equal detection rate). The class comparison analysis was performed using a moderated *t*-test as implemented in the *limma* package.^36^ P-values were adjusted for multiple testing using Benjamini–Hochberg false discovery rate (FDR). The genes with an absolute fold change ⩾2 and FDR⩽0.01 were considered statistically significant. To evaluate the fine-tuning gene deregulation in response to the CCAR2 inactivation a less stringent analysis was performed (FC⩾1.5 and FDR⩽0.05) and the differentially expressed genes were loaded in the Ingenuity Pathway Analysis software (www.ingenuity.com) to point out the deregulated gene networks. Microarray data were deposited and are available on NCBI Gene Expression Omnibus (GEO) database (www.ncbi.nlm.nih.gov/geo/) with the accession number GSE87364.

### RNA extraction and real-time quantitative PCR

For quantitative real-time quantitative PCR (qPCR) total RNA was extracted from U2OS, A549, HME and IOSE80 cells with the mRNeasy mini columns. Overall, 1 *μ*g of total RNA was reverse transcribed using the Transcriptor First Strand c-DNA Synthesis kit (Roche, Basel, Switzerland). real-time qPCR was performed with SYBR Green qPCR Master Mix (Thermo Fisher Scientific) and GAPDH mRNA was used for normalization. Primers used were: TRB3_forward TGCCCTACAGGCACTGAGTA; TRB3_reverse GTCCGAGTGAAAAAGGCGTA; GAPDH_forward AATCCCATCACCATCTTCCA and GAPDH_reverse TGGACTCCACGACGTACTCA.

### Evaluation of G1/S transition

DNA replicating cells were evaluated with the Click-iT EdU assay kit (Thermo Fisher Scientific) as previously described.^37^ Briefly, cells were cultured in EdU containing medium for 1 h and 30 min and then stained according to manufacturer's instructions. Cells positive for EdU were counted and expressed as percentage of the total population.

## Abbreviations

CCAR2: cell cycle and apoptosis regulator 2
DBC1: deleted in breast cancer 1
ERK: extracellular signal-regulated kinase-1
GSK3*β*: glycogen synthase kinase 3 beta
EdU: 5-ethynyl-2′-deoxyuridine
