# Supplementary material for: A novel crosstalk between CCAR2 and AKT pathway in the regulation of cancer cell proliferation
Source: Cell Death Dis. 2016 Nov 3;7(11):e2453–. doi: 10.1038/cddis.2016.359 (PMC5260903; doi:10.1038/cddis.2016.359)
Supplement: Supplementary Figures and Tables [file cddis2016359x1.pdf]

S1

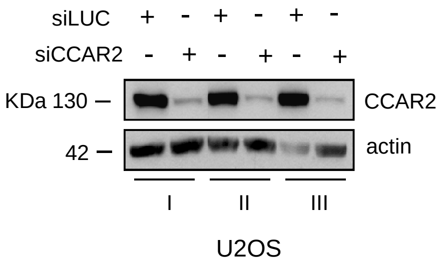

S2

A

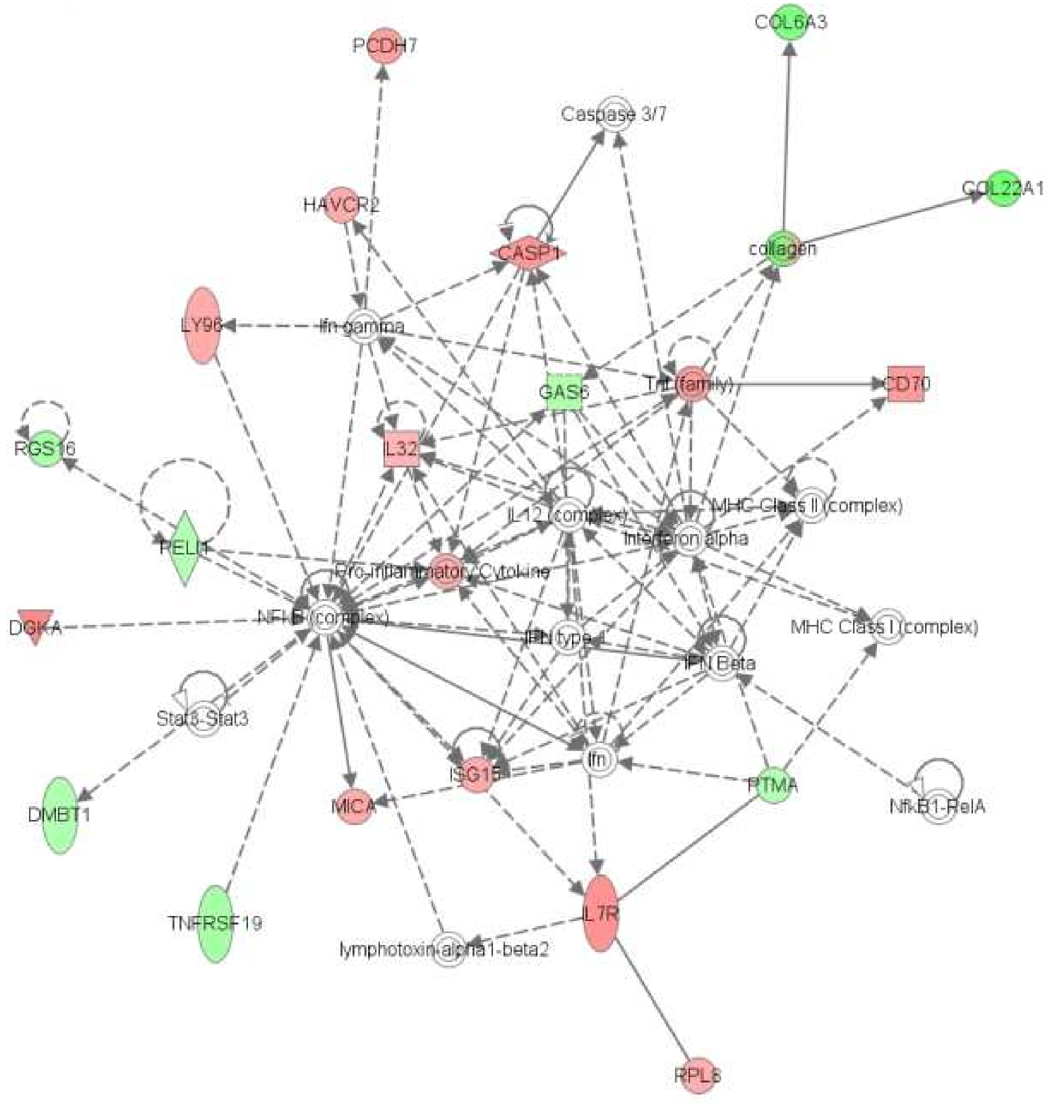
COL6A3
PCDH7
Caspase 3/7
COL22A1
HAVCR2
CASP1
collagen
LY96
Ifn gamma
GAS6
Tnf (family)
CD70
RGS16
IL32
MHC Class II (complex)
IL12 (complex)
Interferon alpha
PELI1
Pro-inflammatory Cytokine
DGKA
NFkB (complex)
IFN type 1
MHC Class I (complex)
IFN Beta
Stat3-Stat3
Ifn
ISG15
PTMA
NfkB1-RelA
DMBT1
MICA
IL7R
TNFRSF19
lymphotoxin-alpha1-beta2
RPL8

N1 (NFkB)

B

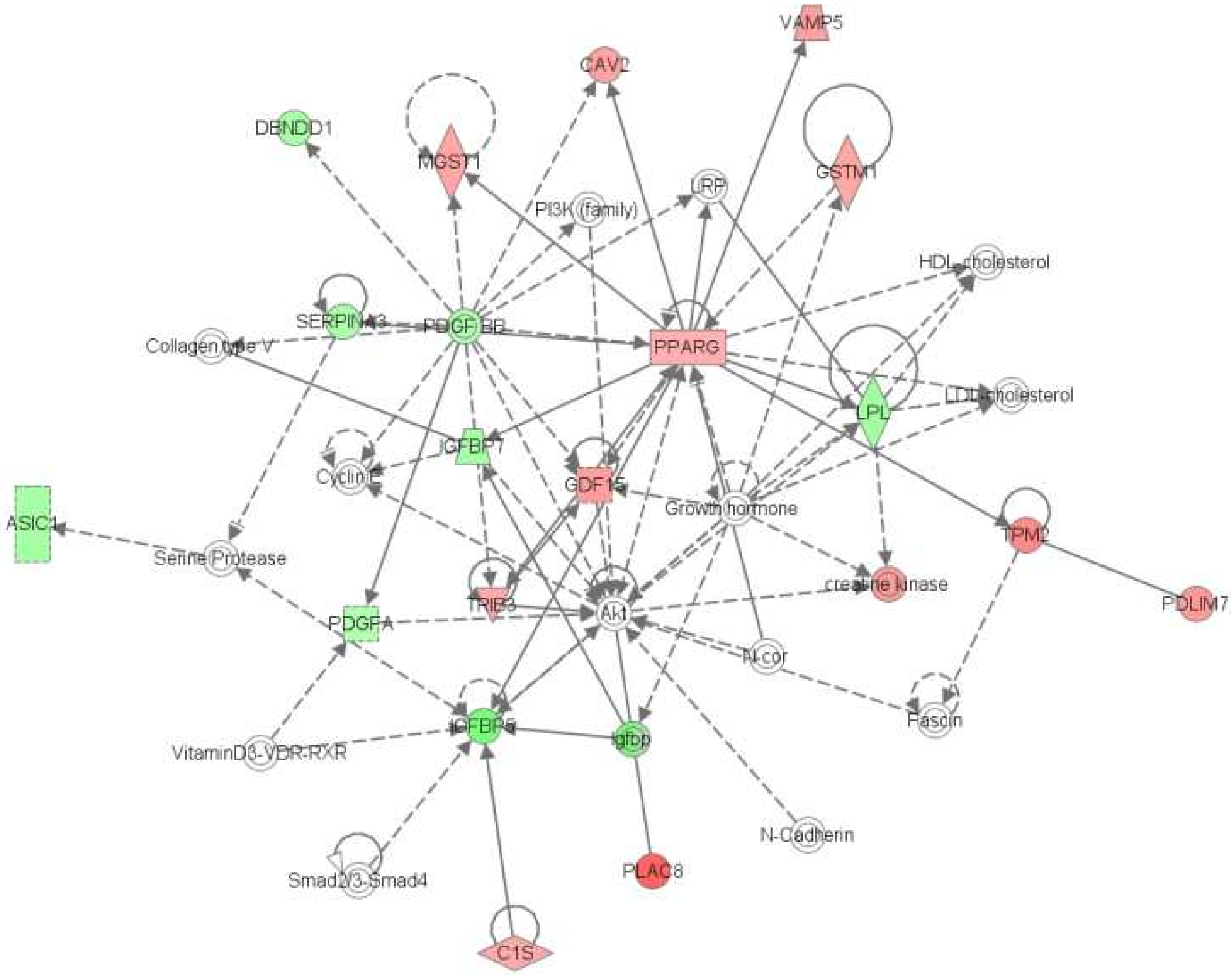
VAMP5
CAV2
DBNDD1
MGST1
GSTM1
LRP
PI3K (family)
HDL-cholesterol
SERPINA3
PDGF BB
PPARG
Collagen type V
LPL
LDL-cholesterol
IGFBP7
Cyclin E
GDF15
Growth hormone
ASIC1
Serine Protease
TPM2
creatine kinase
PDLIM7
TRIB3
Akt
PDGFA
N-cor
Fascin
IGFBP5
Igfbp
VitaminD3-VDR-RXR
N-Cadherin
PLAC8
Smad2/3-Smad4
C1S

N2 (Akt)

C

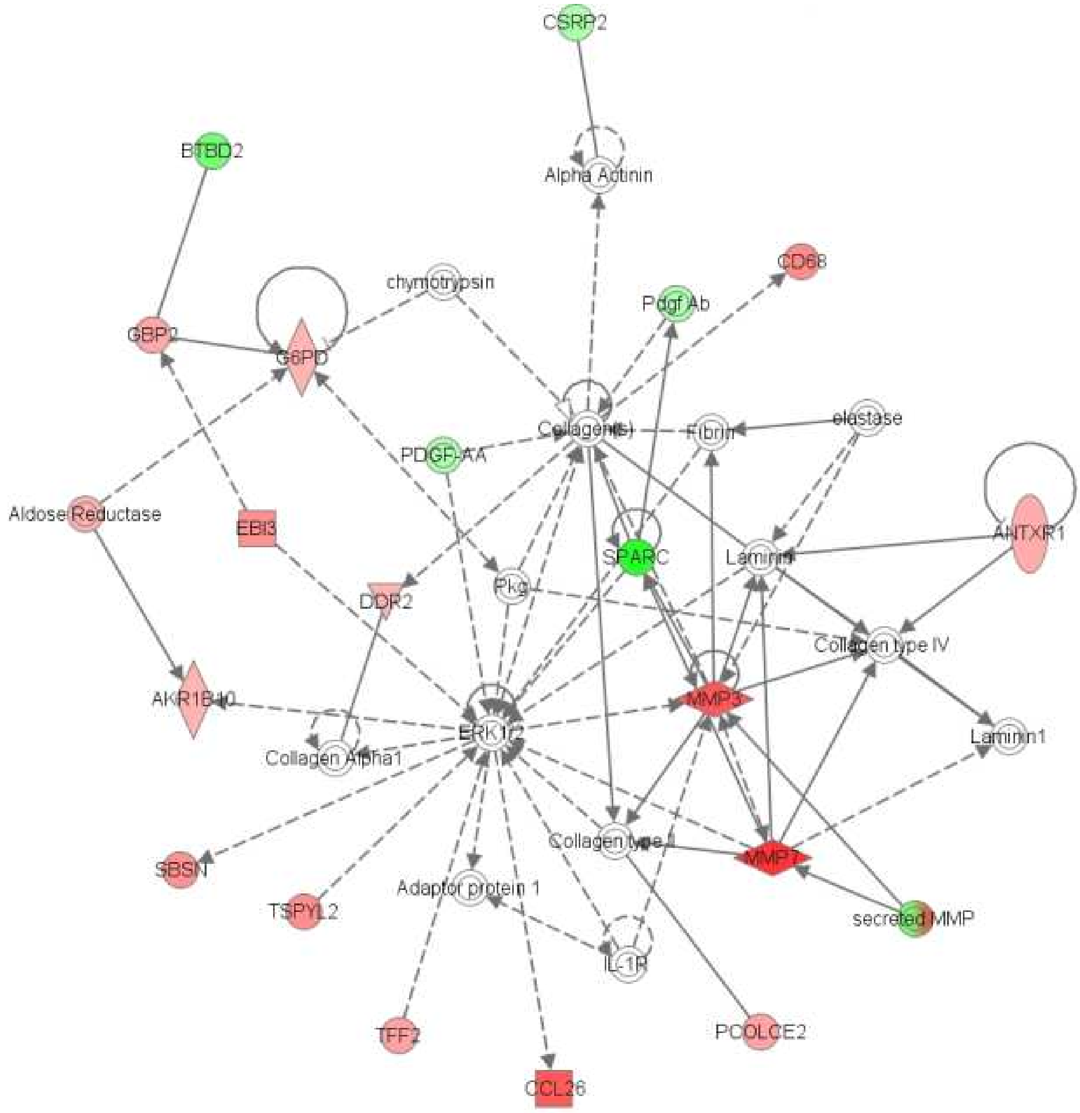
CSRP2
BTBD2
Alpha Actinin
CD68
chymotrypsin
Pdgf Ab
GBP2
G6PD
Collagen(s)
Fibrin
elastase
PDGF-AA
Aldose Reductase
EBI3
SPARC
Laminin
ANTXR1
DDR2
Pkg
Collagen type IV
AKR1B10
MMP3
ERK1/2
Laminin1
Collagen Alpha1
Collagen type I
MMP7
SBSN
TSPYL2
Adaptor protein 1
secreted MMP
IL-1R
TFF2
PCOLCE2
CCL26

N3 (ERK1/2)

D

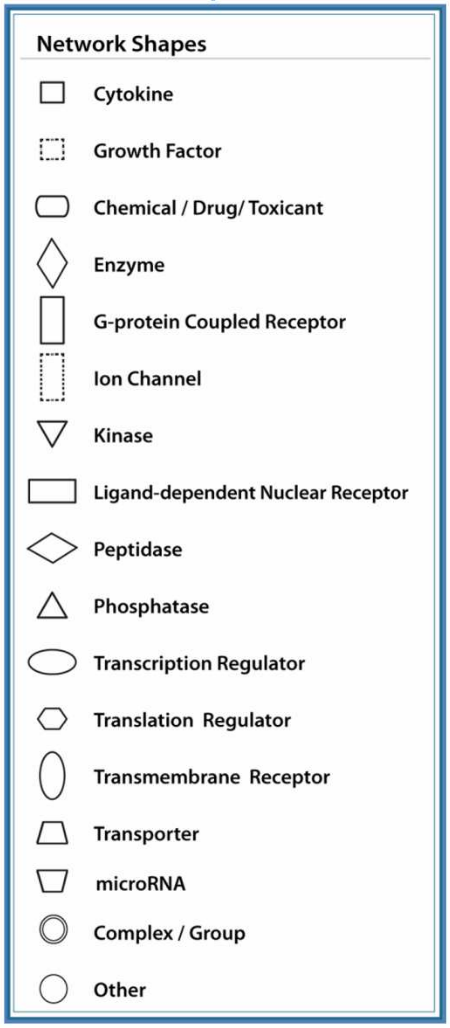
Network Shapes
Cytokine
Growth Factor
Chemical / Drug/ Toxicant
Enzyme
G-protein Coupled Receptor
Ion Channel
Kinase
Ligand-dependent Nuclear Receptor
Peptidase
Phosphatase
Transcription Regulator
Translation Regulator
Transmembrane Receptor
Transporter
microRNA
Complex / Group
Other

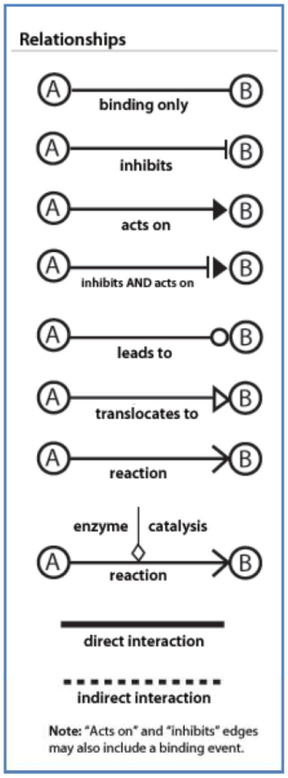
Relationships
binding only
inhibits
acts on
inhibits AND acts on
leads to
translocates to
reaction
enzyme
catalysis
reaction
direct interaction
indirect interaction
Note: "Acts on" and "inhibits" edges may also include a binding event.

S3

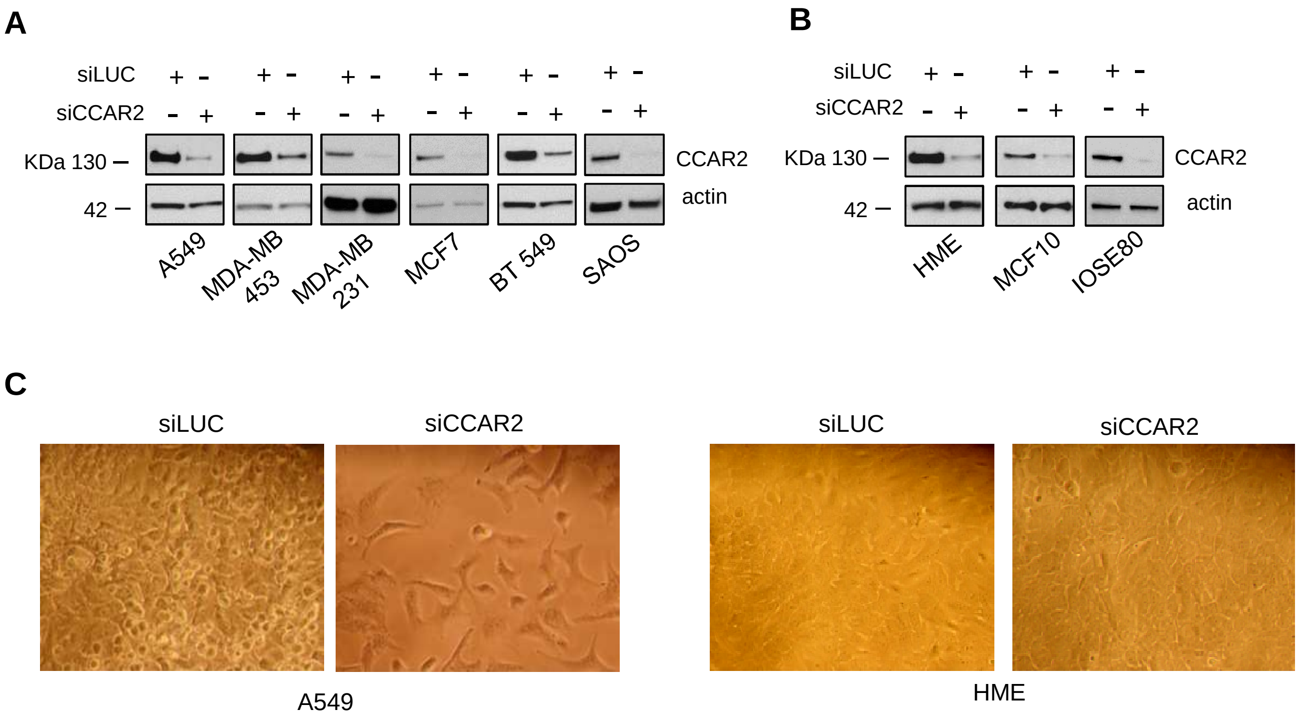
A
siLUC
siCCAR2
KDa 130
42
CCAR2
actin
A549
MDA-MB 453
MDA-MB 231
MCF7
BT 549
SAOS
B
siLUC
siCCAR2
KDa 130
42
CCAR2
actin
HME
MCF10
IOSE80
C
siLUC
siCCAR2
A549
siLUC
siCCAR2
HME

S4

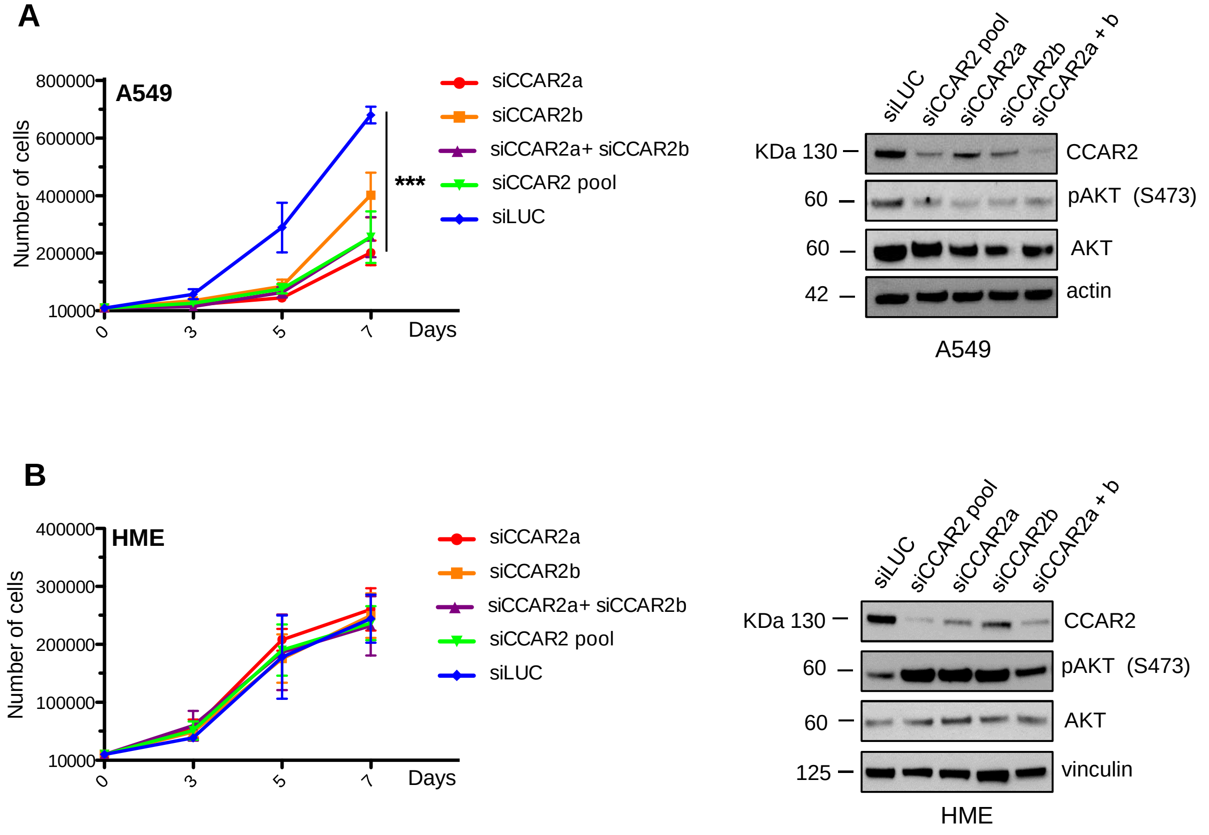
A
A549
Number of cells
800000
600000
400000
200000
10000
0
3
5
7
Days
***
siCCAR2a
siCCAR2b
siCCAR2a+ siCCAR2b
siCCAR2 pool
siLUC
siLUC
siCCAR2 pool
siCCAR2a
siCCAR2b
siCCAR2a + b
KDa 130
60
60
42
CCAR2
pAKT (S473)
AKT
actin
A549
B
HME
Number of cells
400000
300000
200000
100000
10000
0
3
5
7
Days
siCCAR2a
siCCAR2b
siCCAR2a+ siCCAR2b
siCCAR2 pool
siLUC
siLUC
siCCAR2 pool
siCCAR2a
siCCAR2b
siCCAR2a + b
KDa 130
60
60
125
CCAR2
pAKT (S473)
AKT
vinculin
HME

S5

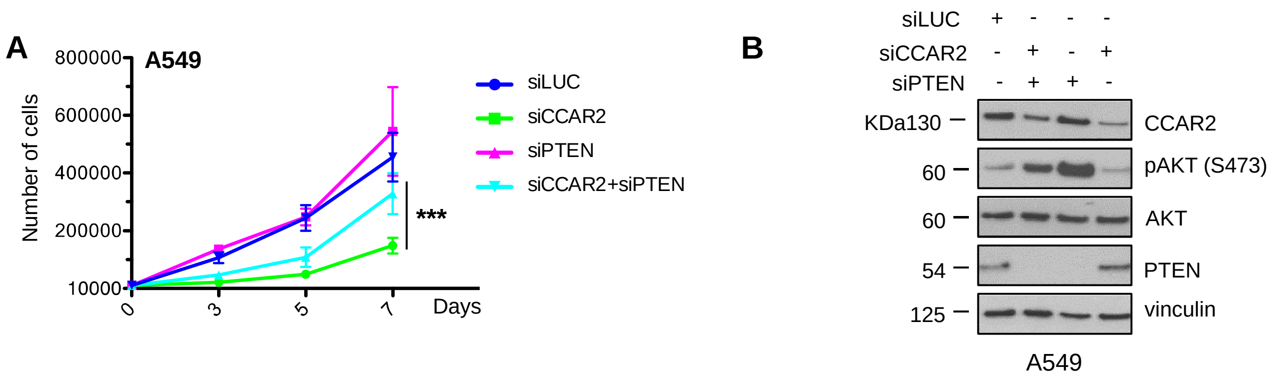
A
A549
800000
600000
400000
200000
10000
Number of cells
0
3
5
7
Days
***
siLUC
siCCAR2
siPTEN
siCCAR2+siPTEN
B
siLUC + - - -
siCCAR2 - + - +
siPTEN - + + -
KDa130
60
60
54
125
CCAR2
pAKT (S473)
AKT
PTEN
vinculin
A549

S6

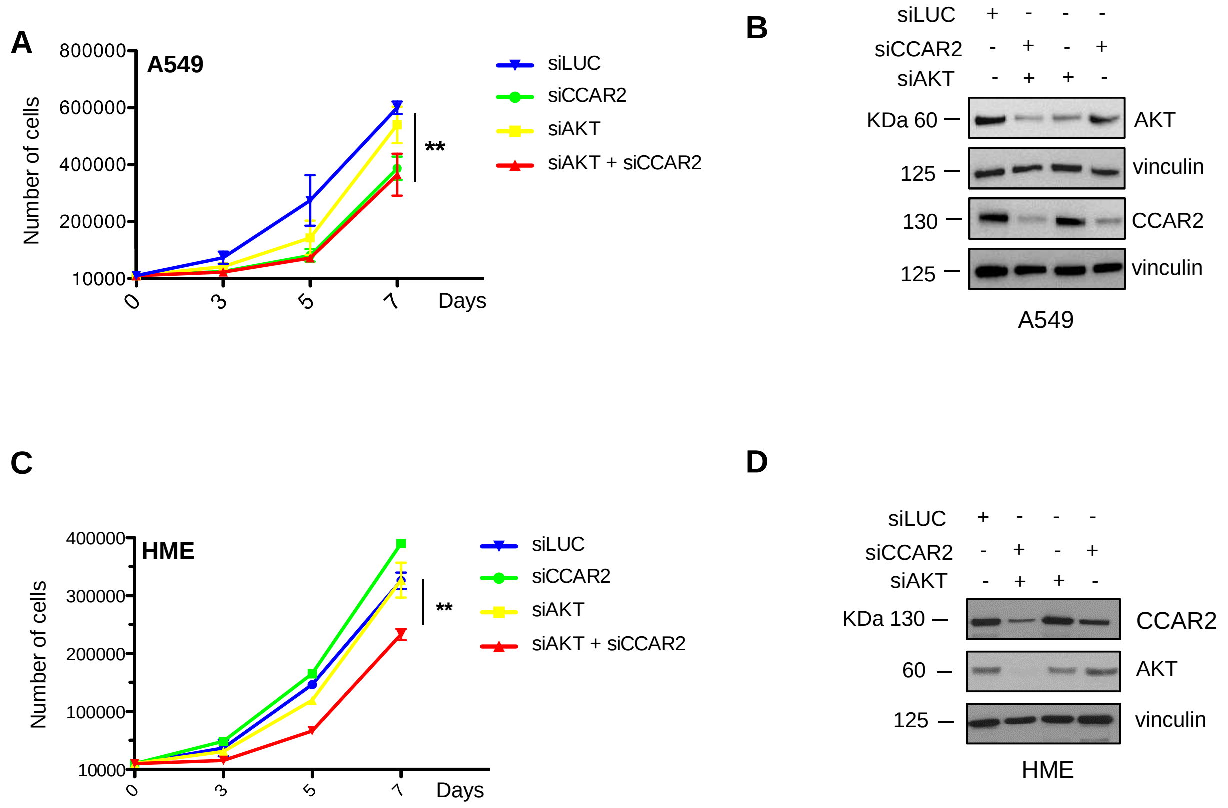
A
A549
800000
600000
400000
200000
10000
Number of cells
0
3
5
7
Days
**
siLUC
siCCAR2
siAKT
siAKT + siCCAR2
B
siLUC + - - -
siCCAR2 - + - +
siAKT - + + -
KDa 60
125
130
125
AKT
vinculin
CCAR2
vinculin
A549
C
HME
400000
300000
200000
100000
10000
Number of cells
0
3
5
7
Days
**
siLUC
siCCAR2
siAKT
siAKT + siCCAR2
D
siLUC + - - -
siCCAR2 - + - +
siAKT - + + -
KDa 130
60
125
CCAR2
AKT
vinculin
HME

S7

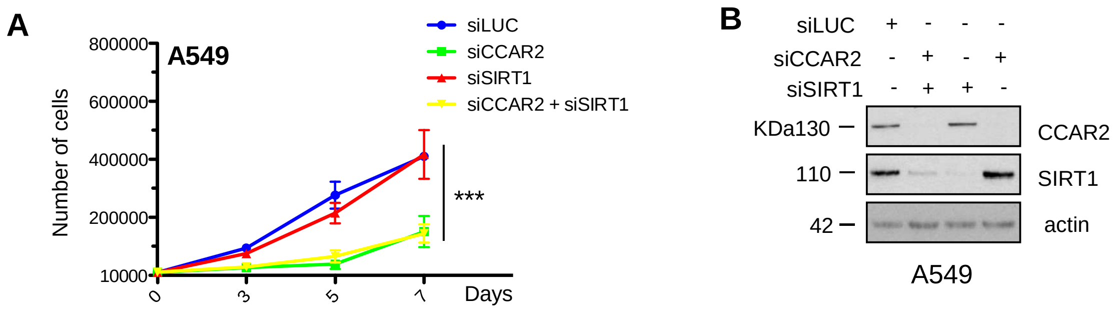
A
A549
800000
600000
400000
200000
10000
Number of cells
0
3
5
7
Days
siLUC
siCCAR2
siSIRT1
siCCAR2 + siSIRT1
***
B
siLUC + - - -
siCCAR2 - + - +
siSIRT1 - + + -
KDa130
110
42
CCAR2
SIRT1
actin
A549

S8

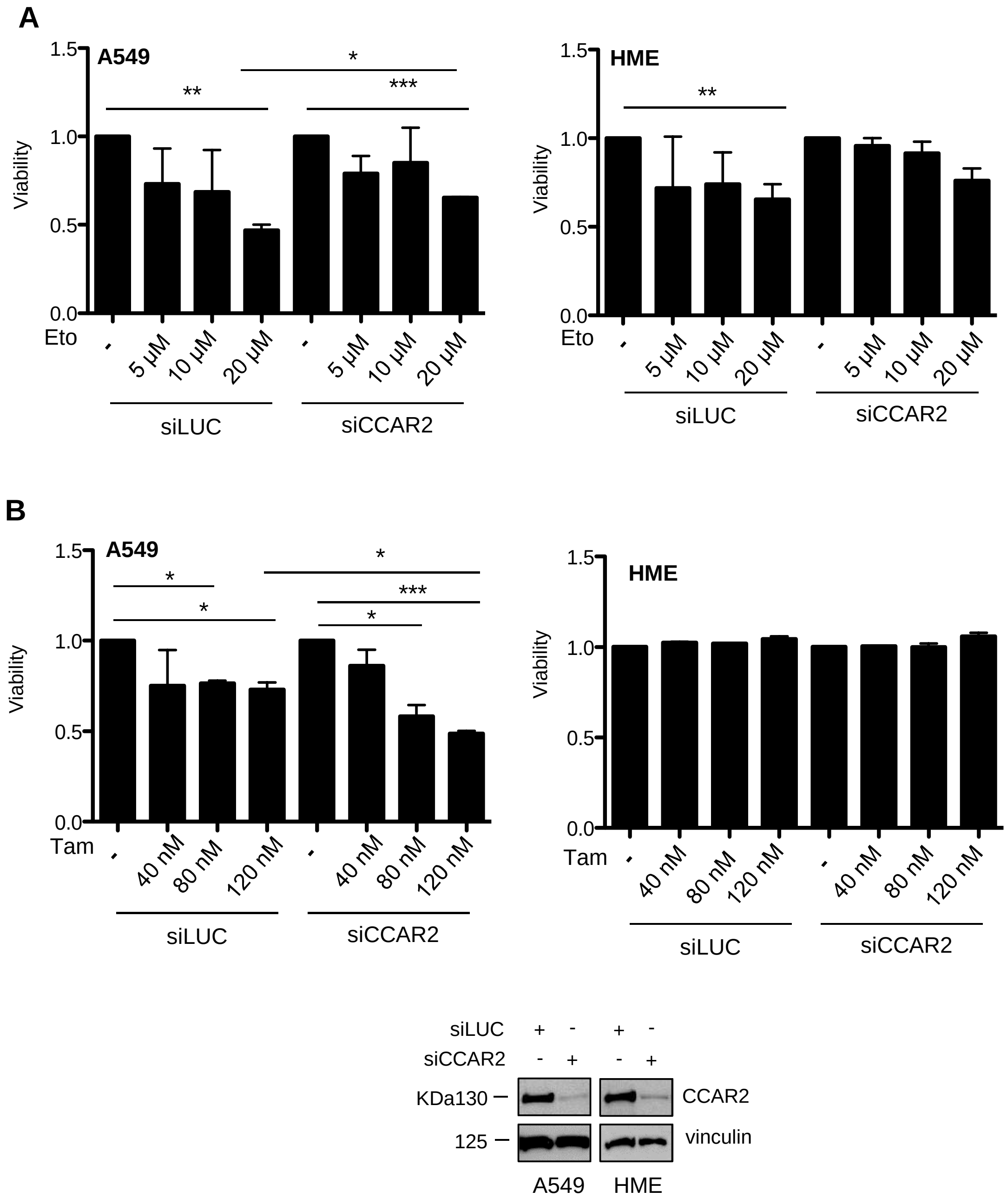
A
A549
HME
Viability
1.5
1.0
0.5
0.0
Eto
-
5 μM
10 μM
20 μM
siLUC
siCCAR2
*
**
***
B
Tam
40 nM
80 nM
120 nM
siLUC
siCCAR2
siLUC + - + -
siCCAR2 - + - +
KDa130
125
CCAR2
vinculin
A549
HME

S9

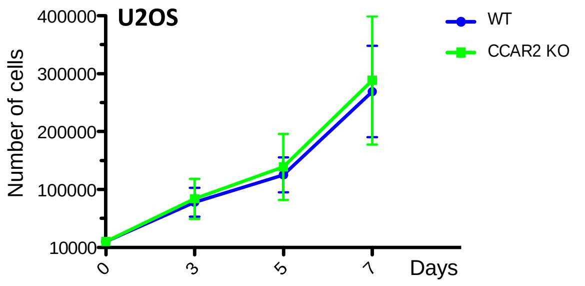
U2OS
Number of cells
400000
300000
200000
100000
10000
0
3
5
7
Days
WT
CCAR2 KO

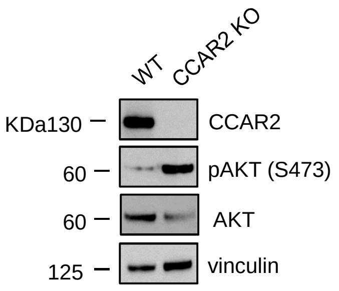
WT
CCAR2 KO
KDa130
60
60
125
CCAR2
pAKT (S473)
AKT
vinculin

S10

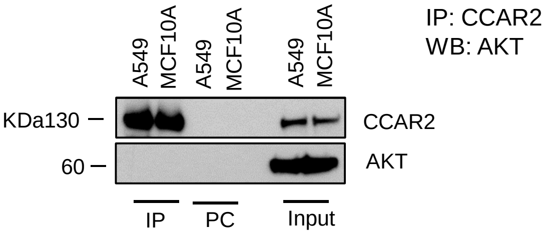
A549
MCF10A
A549
MCF10A
A549
MCF10A
IP: CCAR2
WB: AKT
KDa130
60
CCAR2
AKT
IP
PC
Input

S11

A

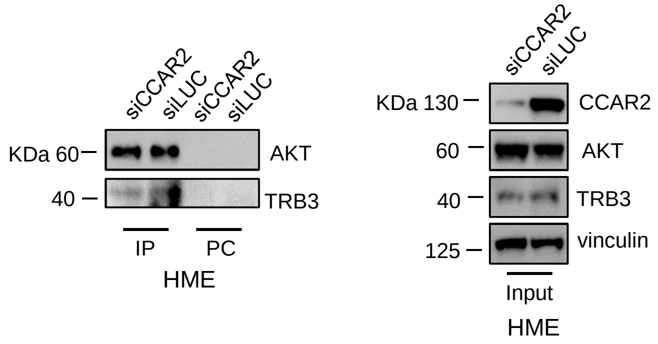
siCCAR2
siLUC
siCCAR2
siLUC
KDa 60
40
AKT
TRB3
IP
PC
HME
siCCAR2
siLUC
KDa 130
60
40
125
CCAR2
AKT
TRB3
vinculin
Input
HME

B

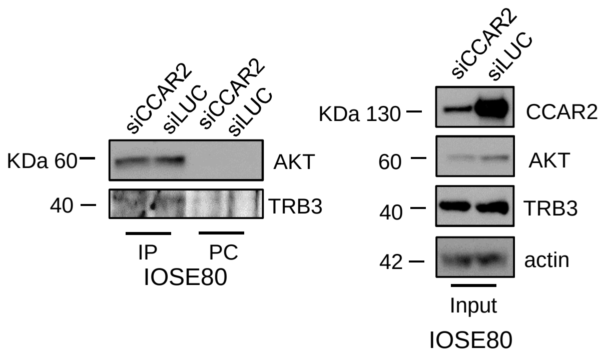
siCCAR2
siLUC
siCCAR2
siLUC
KDa 60
40
AKT
TRB3
IP
PC
IOSE80
siCCAR2
siLUC
KDa 130
60
40
42
CCAR2
AKT
TRB3
actin
Input
IOSE80

Supplementary Table 2

Name	p-Value	Molecules
Cellular Growth and Proliferation	3.21E-10 - 2.10E-03	72
Cellular Development	6.07E-08 - 2.26E-03	69
Cell Death and Survival	4.41E-07 - 2.26E-03	63
Cellular Movement	1.04E-06 - 1.73E-03	45
Lipid Metabolism	3.89E-06 - 1.80E-03	33

Supplementary Table 4

A

	p53
**U2OS**	WT
**SAOS2**	null
**A549**	WT
**MDA-MB-453**	mut
**MDA-MB-231**	mut
**MCF-7**	WT
**BT-549**	mut

B

	ER status
**MDA-MB-453**	neg
**MDA-MB-231**	neg
**MCF-7**	pos
**BT-549**	neg
