# Supplementary material for: A novel crosstalk between CCAR2 and AKT pathway in the regulation of cancer cell proliferation
Source: Cell Death Dis. 2016 Nov 3;7(11):e2453–. doi: 10.1038/cddis.2016.359 (PMC5260903; doi:10.1038/cddis.2016.359)
Supplement: Supplementary Information [file cddis2016359x4.pdf]

SUPPLEMENTARY FIGURE and TABLE LEGENDS

Figure S1

WB analysis of U2OS cells transfected with siCCAR2 and siLUC and harvested 6 days after transfection for gene expression analysis. The experiment was repeated in triplicate.

Figure S2

Graphical representation of the N1, N2 and N3 (**A**, **B** and **C** respectively) networks identified by IPA. The network is displayed graphically as nodes (genes) and arrows (the biological relationships between nodes). The node color intensity is related to changes in gene expression levels (red, up-regulated; green, down-regulated genes). The figure legend (**D**) describes the meaning of network shapes and their relationships.

Figure S3

A) WB analysis of CCAR2 protein levels in the indicated cancer cell lines transfected with siCCAR2 and siLUC and used for cellular proliferation assays. **B)** WB analysis of the indicated normal cell lines transfected with siCCAR2 and siLUC and analyzed as above. **C)** Representative images of A549 and HME cells transfected with siCCAR2 and siLUC, seeded at 10000 cells/well and cultured for 7 days.

Figure S4

A) Cell proliferation rate (left) and WB analysis (right) of A549 cells silenced with 2 different

siCCAR2 sequences (siCCAR2a and siCCAR2b), their combination (siCCAR2a+siCCAR2b) and the iBONi siRNA pool of four sequences (siCCAR2 pool). All the sequences used caused a strong reduction of cell proliferation and levels of phosphorylated AKT. **B)** Cell proliferation rate (left) and WB analysis (right) of HME cells silenced with 2 different siCCAR2 sequences (siCCAR2a and siCCAR2b), their combination (siCCAR2a+siCCAR2b) and the iBONi siRNA pool of four sequences (siCCAR2 pool). All the sequences used do not impact on cell proliferation and all induce AKT activation.

Figure S5

A) Cell proliferation rate in A549 cells silenced for CCAR2, PTEN or both CCAR2 and PTEN (siLUC was used as negative control). CCAR2 silencing caused a reduction in cancer cell proliferation, PTEN silencing did not affect cancer cell proliferation, while double silencing of CCAR2 and PTEN partially rescued the proliferation defects caused by CCAR2 depletion. **B)** WB analysis with the indicated antibodies of A549 cells transfected with siRNA against CCAR2, PTEN and both siCCAR2 and siPTEN, siLUC was used as negative control.

Figure S6

A) Cell proliferation rate in A549 cells silenced for CCAR2, AKT or both CCAR2 and AKT (siLUC was used as negative control). CCAR2 silencing caused a reduction in cancer cell proliferation, AKT silencing modestly decreased cell proliferation, while cells double silenced for AKT and CCAR2 behaved like cells silenced for CCAR2. **B)** Western blot analysis with the indicated antibodies of A549 cells transfected with siRNA against CCAR2, AKT and both siCCAR2 and

siAKT, siLUC was used as negative control. **C)** Cell proliferation rate in HME cells silenced for CCAR2, AKT or both CCAR2 and AKT (siLUC was used as negative control). **D)** WB analysis with the indicated antibodies of HME cells transfected with siRNA against CCAR2, AKT and both siCCAR2 and siAKT, siLUC was used as negative control.

Figure S7

A) Cell proliferation rate in A549 cells silenced for CCAR2, SIRT1 or both CCAR2 and SIRT1 (siLUC was used as negative control). CCAR2 silencing caused a reduction in cancer cell proliferation, SIRT1 silencing did not significantly alter cell proliferation and cells double silenced for SIRT1 and CCAR2 behaved like the cells silenced for CCAR2, indicating that SIRT1 and CCAR2 does not act together in the regulation of cell proliferation. **B)** WB analysis with the indicated antibodies of A549 cells transfected with siRNA against CCAR2, SIRT1 and both siCCAR2 and siSIRT1, siLUC was used as negative control.

Figure S8

A) A549 (left) and HME (right) cell lines silenced for CCAR2 were treated with increasing amounts of Etoposide drug. Cell viability was measured using CellTiter-Glo® Luminescent assay**.**
B) A549 (left) and HME (right) cell lines silenced for CCAR2 were treated with increasing amounts of Tamoxifen drug. Cell viability was measured using CellTiter-Glo® Luminescent assay.

Figure S9

Cell proliferation rate (left) and WB analysis (right) of U2OS WT and U2OS CCAR2-KO cells. The

U2OS CCAR2-KO cell line does not display any statistically significant proliferation defect whereas CCAR2 absence caused a strong increase of pAKT.

Figure S10

Total protein extracts from A549 and MCF10A cells were immunoprecipitated with an anti CCAR2 antibody and then analysed by WB with the indicated antibody. Pre-clearing was used as a negative control for the IP, Input is shown on the right.

Figure S11

AKT immunoprecipitates from HME **(A)** and IOSE80 **(B)** cells silenced for CCAR2 and LUC were analysed by WB with the indicated antibodies. Pre-clearing was used as a negative control for the IP, Input is shown on the right.

Table 1

List of genes whose expression is significantly altered in U2OS cells silenced for CCAR2.

Table 2

Table reporting the networks identified by IPA software.

Table 3

List of genes whose expression is significantly altered in BJ-hTERT cells silenced for CCAR2.

Table 4

Tables indicating the p53 status **(A)** in all the cell lines tested and ER status **(B)** in all the breast cancer cell lines used.
